# Implementing tin-prefiltration in routine clinical CT scans of the lower extremity: impact on radiation dose

**DOI:** 10.1007/s00256-025-04897-3

**Published:** 2025-02-26

**Authors:** Thomas Marth, Georg Wilhelm Kajdi, Christoph Stern, Reto Sutter

**Affiliations:** 1 Swiss Center for Musculoskeletal Imaging, Balgrist Campus AG, Zurich, Switzerland; 2 Department of Radiology, Balgrist University Hospital, Zurich, Switzerland; 3 Medical Faculty, University of Zurich, Zurich, Switzerland

**Keywords:** CT, Musculoskeletal imaging, Tin filter, Radiation dose, Lower extremity

## Abstract

**Objectives:**

Several studies have demonstrated the potential of tin-prefiltration to reduce radiation dose while maintaining diagnostic image quality for musculoskeletal imaging. Still, no study has reported data on the impact of tin-prefiltration on radiation dose reduction for clinical routine scanning.

**Materials and methods:**

Retrospective inclusion of 300 clinically indicated CT scans of the pelvis, knee, and ankle before January 2020 (without tin filter) and after December 2020 (with tin filter). For each joint, 50 examinations with tin-prefiltration and 50 examinations without tin-prefiltration were selected. Dose parameters were extracted, calculated, and compared. Subjective and quantitative parameters for image quality were assessed.

**Results:**

The CTDI_vol_, DLP, and effective dose were reduced significantly in all tin-prefiltered examinations compared to the non-tin-prefiltered examinations (*p* < 0.001): CTDI_vol_ was 65% lower in the pelvis, 73% lower in the knee, and 54% lower in the ankle. This reduced the effective dose of 61%, 71%, and 60%, respectively. In absolute numbers, the reduction of the median effective dose delivered in a single CT scan of the pelvis was − 2.29 mSv, − 0.15 mSv for the knee, and − 0.03 mSv for the ankle. No difference in diagnostic image quality, depiction of bone anatomy and soft tissues, and image artifacts was observed (*p* > 0.05). Subjective and objective image noise was higher in tin-prefiltered pelvis CT (*p* < 0.001).

**Conclusion:**

The implementation of tin-prefiltration in clinical routine scan protocols significantly reduced the effective radiation dose for unenhanced CT scans of the lower extremities between 60 and 70%.

**Supplementary Information:**

The online version contains supplementary material available at 10.1007/s00256-025-04897-3.

## Introduction

Ionizing radiation is known for its deterministic and stochastic effects in the body, with the first being observed above a specific threshold dose, while the latter can occur even at the lowest radiation doses, but nevertheless have a risk to induce radiation-induced cancer [1, 2]. The latter is conventionally referred to as the linear no-threshold (LNT) model and is based on the assumption that the radiation-related risk of stochastic effects is directly proportional to the dose received and that there is no dose threshold below which there is no risk. The model is still being debated with several forms of dose–effect relationships being discussed at low doses, supporting a possible existence of a dose threshold below which there is no risk or even models estimating health benefits (hormesis effect) [3]. Although the effects of low radiation doses are debated, the LNT model still serves as a prudent basis for the practical purposes of current radiological protection systems [2]. While the risk estimation for low radiation exposure remains uncertain, the radiation doses delivered in computed tomography (CT) examinations, ranging from less than 1 mSv to around 10 mSv, have the potential to induce stochastic effects. Therefore, a conservative approach is recommended, keeping doses of ionizing radiation “as low as reasonably achievable” following the “ALARA” principle and using a linear no-threshold dose–response model [2, 4]. To bring the radiation dose delivered by CT examinations in context, it is helpful to look at the annual radiation dose exposure per person per year, which is estimated to 6 mSv in the swiss population and 6.3 mSv in the USA [5, 6]. Since CT examinations contribute 64.3% of the annual 1.49 mSv medical radiation exposure in the swiss population, they make up the largest share while only being the third most used imaging modality with a frequency of 11%. Therefore, a particular focus lies on the dose reduction in every single CT scan [5, 7]. Different radiation dose reduction strategies in CT imaging are currently being pursued, and one of them is focusing on prefiltration of the X-ray beam using a tin filter [8]. When this tin filter is positioned in front of the X-ray tube, it selectively filters out low-energy photons of the X-ray beam before they reach the patient. These low-energy photons contribute substantially to the radiation dose but not to the image quality of unenhanced CT scans with a focus on inherently high-contrast structures such as the bone or the lung [9, 10]. With tin-prefiltration, the resulting hardened, or spectral-shaped energy spectrum of the X-ray beam has a higher mean photon energy with a higher percentage of X-ray photons that penetrate the patient, reach the detector, and contribute to bone imaging [9]. Several studies demonstrated the potential of radiation dose reduction of tin-prefiltration while maintaining diagnostic image quality for various diagnostic imaging tasks with focus on high contrast structures [8, 11–16].

Still, no study has reported data on the impact of tin-prefiltration on radiation dose reduction for musculoskeletal CT in clinical routine. Therefore, we set out to compare CT dose index volume (CTDI_vol_), dose length product (DLP), and effective dose before and after implementing the tin filter in clinical routine musculoskeletal CT imaging for the pelvis, knee, and ankle.

## Materials and methods

### Ethics approval

This retrospective single-center study was approved by the local institutional review board (cantonal ethics committee Zurich). It was performed according to the principles of the Declaration of Helsinki and national ethics standards. All patients signed a written general consent form allowing their health-related data to be used for research purposes.

### Patients

**Fig. 1:**
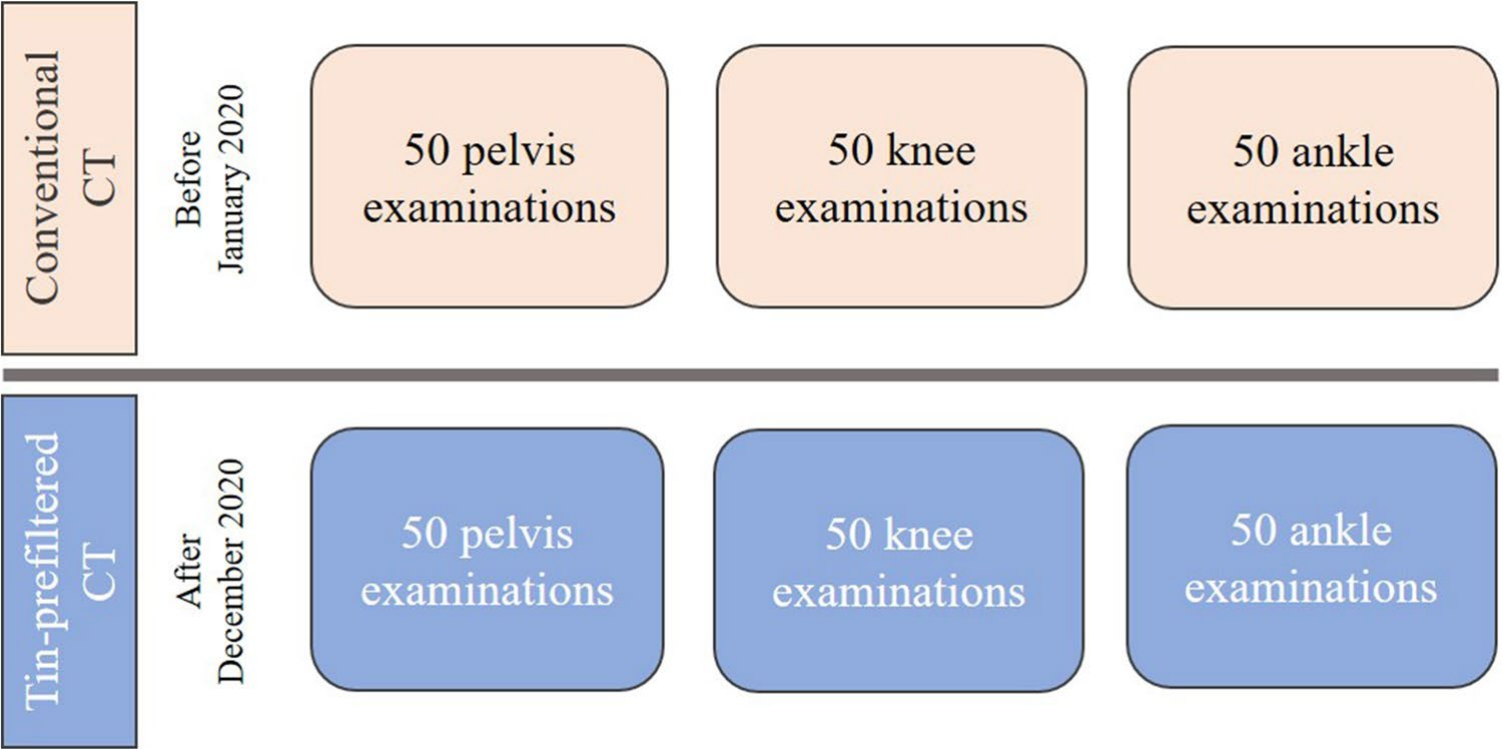
Schematic overview of the 300 evaluated examinations. For each scan location, 100 CT scans were included, equally partitioned into 50 examinations with tin filter and 50 examinations without tin filter

For retrospective identification of the patient cohorts, the institutional radiation monitoring system (EasyDose-QM, version 1.8.131, BMS) was searched for patients who underwent clinically indicated CT scans of the pelvis, knee, or ankle on a single CT scanner (SOMATOM Definition AS/AS + , Siemens Healthineers) before January 2020 (without tin filter) and after December 2020 (with tin filter). Only patients who provided written informed consent were included in the study. Patients with metal implants in the scan area and patients under the age of 18 years were excluded. In total, 300 examinations from 288 patients were included. For each joint (hip, knee, ankle), 50 examinations with tin-prefiltration and 50 examinations without tin-prefiltration were selected (Fig. 1). Patient characteristics such as age, body mass index (BMI), and gender were extracted from the picture archiving and communication system (Merlin, Phoenix-PACS) and the electronic patient records.

### CT examinations

All images were acquired in the clinical setting and conducted using the standard clinical protocol in the corresponding period. All examinations were performed on the same CT scanner (SOMATOM Definition AS/AS + , Siemens Healthineers) before and after installation of the tin filter. All patients were imaged in a supine position. The pelvis, knee, and ankle examinations were checked within the PACS for metal implants in the scan region. If present, the examinations were excluded to allow a better comparison between the groups. For every single scan, radiation dose parameters CTDI_vol_, DLP, and effective dose, as well as the acquisition parameters kV, mAs/rotation, collimation, pitch factor, and scan length, were extracted from EasyDose-QM and PACS. For better comparison of qualitative and quantitative image analysis, only images reconstructed with the same convolution kernel, slice thickness, and iterative reconstruction strength were evaluated. Four scans with differing reconstruction parameters were excluded from qualitative and quantitative image analysis, one examination reconstructed with BR38 in the conventional group in the knee, and three examinations reconstructed with BR51 in the tin-prefiltered group in the knee. The CT acquisition and reconstruction parameters are listed in the expanded Table 1.

**Table 1:** CT acquisition and reconstruction parameters

Pelvis	Conventional CT	Tin-prefiltered CT
Scanner model	SOMATOM Definition AS	SOMATOM Definition AS +
Tube potential (kVp)	120	140
mAs/rotation	119.5 (119.5–119.5)	142 (142–142)
Collimation (mm)	32 × 0.6	64 × 0.6
Pitch factor	0.8	0.8
Rotation time (s)	0.5	1
Exposure modulation type	None	None
Scan length (mm)	243.5 (226–261.2)	322 (310–337)
Field of view (mm × mm)	500 × 500	500 × 500
Matrix	512 × 512	512 × 512
Axial source slice thickness (mm)	1	0.5
Increment	0.5	0.5
Reconstruction kernel	BR38	BR38
Iterative reconstruction strength	3	3
Tin-prefiltration	No	Yes
Knee	Conventional CT	Tin-prefiltered CT
Scanner model	SOMATOM Definition AS	SOMATOM Definition AS +
Tube potential (kVp)	120	100
mAs/rotation	95.5 (95–96)	305 (291–319)
Collimation (mm)	32 × 0.6	64 × 0.6
Pitch factor	0.8	0.5
Rotation time (s)	0.5	1
Exposure modulation type	None	Angular modulation XY
Scan length (mm)	375 (375–376)	381 (381–382)
Field of view (mm × mm)	200 × 200	200 × 200
Matrix	512 × 512	512 × 512
Axial source slice thickness (mm)	1	1
Increment	0.5	1
Reconstruction kernel	BR59	BR59
Iterative reconstruction strength	3	3
Tin-prefiltration	No	Yes
Ankle	Conventional CT	Tin-prefiltered CT
Scanner model	SOMATOM Definition AS	SOMATOM Definition AS +
Tube potential (kVp)	120	100
mAs/rotation	71 (69–72)	379 (374–383)
Collimation (mm)	32 × 0.6	64 × 0.6
Pitch factor	0.8	0.5
Rotation time(s)	1	1
Exposure modulation type	Angular Modulation XY	Angular Modulation XY
Scan length (mm)	183.5 (169–198.5)	184 (172–214.7)
Field of view (mm × mm)	151 (140–181)	164 (148–177)
Matrix	512 × 512	512 × 512
Axial source slice thickness (mm)	0.6	0.6
Increment	0.4	0.4
Reconstruction kernel	BR62	BR62
Iterative reconstruction strength	3	3
Tin-prefiltration	No	Yes

mAs/rotation and scan length are reported as median, with 25th percentile (Q1) and 75th percentile (Q3) in parentheses (Q1–Q3)

### Quantitative analysis of radiation dose parameters and patient characteristics

All CTDI_vol_ estimates were computed by the scanner using the 32 cm IEC Body Dosimetry Phantom as reference. The effective dose displayed in EasyDose-QM was calculated by the integrated software VirtualDose™CT (Virtual Phantoms Inc.). The percentage difference in median CTDI_vol_, DLP, and effective dose between tin-prefiltered and conventional scans was computed. Tin-prefiltered and conventional scans were compared for each location regarding CTDI_vol_, DLP, effective dose, and scan length using the Mann–Whitney U test. The body mass index (BMI) was computed manually based on the extracted patient’s height and weight. Patient characteristics such as age and BMI were compared using the Mann–Whitney U or the *t*-test. Patients’ gender was compared using the Chi-square test.

### Qualitative image analysis

Two musculoskeletal radiologists (T.M. with 4 years of experience and G.W. K with 8 years of experience) rated conventional and tin-prefiltered CT images on a PACS workstation and were blinded to each other. Images were anonymized and presented in random order. No washout period was necessary as no patient received a conventional and a tin-prefiltered CT of the same body region. Observers used axial, sagittal, and coronal images with the images being displayed in the bone window and additionally in the soft tissue window for evaluation of soft tissue structures. Readers were blinded to clinical information and imaging results. The following features were rated on a 4-point Likert scale: depiction of bone anatomy (1 = poor, 2 = fair, 3 = moderate, 4 = good), depiction of soft tissue (1 = poor, 2 = fair, 3 = moderate, 4 = good), image noise (1 = very high, 2 = high, 3 = moderate, 4 = minimal), image artifacts (1 = very strong, 2 = strong, 3 = weak, 4 = none), and diagnostic image quality (1 = poor, 2 = fair, 3 = moderate, 4 = good). For each reader, scores for each qualitative feature were compared between conventional CT and tin-prefiltered CT examinations using the Mann–Whitney U test. Interreader agreement for the qualitative measures was assessed using weighted kappa coefficients.

### Quantitative image analysis

For objective image assessment, CT values (HU) and standard deviation (SD) of the cortical bone, the muscle, and the subcutaneous fat were evaluated by drawing standardized regions of interest (ROIs) of equal size (10 mm^2^) in nearly identical anatomical locations using anatomical landmarks. Assessment was conducted on axial slice images with a slice thickness of 1 mm in the pelvis, 1 mm in the knee, and 0.6 mm in the ankle. For the cortical bone, ROIs were placed in the cortical bone of the proximal femur (pelvis), the distal femur (knee), and the distal tibia (ankle). For the muscle, ROIs were placed in the gluteus medius muscle (pelvis), the vastus medialis muscle (knee), and the tibialis anterior muscle (ankle). For subcutaneous fat, ROIs were placed in the subcutaneous fat of the lower abdominal wall (pelvis), the anterior subcutaneous fat of the distal femur (knee), and the anterior subcutaneous fat of the distal tibia (ankle). Image noise was defined as the SD of the CT attenuation in the air [10, 14] measured by standardized ROIs of 10 mm^2^ outside the body, anterior to the pelvis wall (pelvis), anterior to the knee (knee), and medial to the distal tibia (ankle). Signal-to-noise ratio (SNR) and contrast-to-noise ratio (CNR) were calculated for cortical bone, muscle, and subcutaneous fat for conventional CT and tin-prefiltered CT. The following equations were used: SNR_tissue_ = (HU_mean_tissue_/SD_background_air_); CNR_tissue1 – tissue2_ = (HU_mean_tissue1_- HU_mean_tissue2_/SD_tissue1_). Quantitative measurements were compared between conventional CT and tin-prefiltered CT examinations using the Mann–Whitney U test.

### Statistical analysis

Continuous variables were assessed for normal distribution with the Shapiro–Wilk test and for homogeneity of variances with the Levene test. If distributed normally, continuous data are presented as mean with standard deviation (SD). Continuous data with non-normal distribution are reported as median with 25th percentile (Q1) and 75th percentile (Q3). Comparisons were considered statistically significant at an alpha level of less than 0.05. Weighted kappa was categorized according to the system of Landis and Koch [17]: less than 0.200, slight agreement; 0.200–0.399, fair; 0.400–0.599, moderate; 0.600–0.799, substantial; and 0.800 or greater, almost perfect. All statistical analyses were performed using SPSS Statistics (version 29, IBM).

## Results

### Patient characteristics

Patient characteristics are reported in Table 2. There was no significant difference in BMI between the conventional CT group and the tin-prefiltered CT group for the pelvis (*p* = 0.06), the knee (*p* = 0.96), and the ankle (*p* = 0.55). There was no significant difference in age between the two groups for the pelvis (*p* = 0.23) and the knee (*p* = 0.54). A significant difference was observed in the ankle in age between the two groups (*p* = 0.02; mean 44.9 (± 15.4) to 52.1 (± 15.9) years). No significant difference in gender was found between conventional CT scans and tin-prefiltered CT scans for the pelvis (*p* = 0.42), for the knee (*p* = 0.32), and for the ankle (*p* = 0.42).

**Table 2:** Patient characteristics

Location		Conventional CT	Tin-prefiltered CT	*p*
Pelvis	BMI (kg/m^2^)	28.5 (± 4.7)	26.6 (± 3.7)	0.06
	Age (y)	66.5 (± 7.8)	63.2 (± 13.6)	0.23
	Male/female	19/31	23/27	0.42
Knee	BMI (kg/m^2^)	27.1 (± 5.1)	27.2 (± 5.5)	0.96
	Age (y)	35.3 (± 15.2)	32.9 (± 12.8)	0.54
	Male/female	28/22	23/27	0.32
Ankle	BMI (kg/m^2^)	27.2 (± 4.7)	27.9 (± 5.9)	0.55
	Age (y)	44.9 (± 15.4)	52.1 (± 15.9)	0.02
	Male/female	24/26	28/22	0.42

Values for BMI and age are reported as mean with standard deviation (± SD) in parentheses in the pelvis, knee, and ankle. Additionally, the number of male and female patients is given

### CT radiation dose parameters

**Fig. 2:**
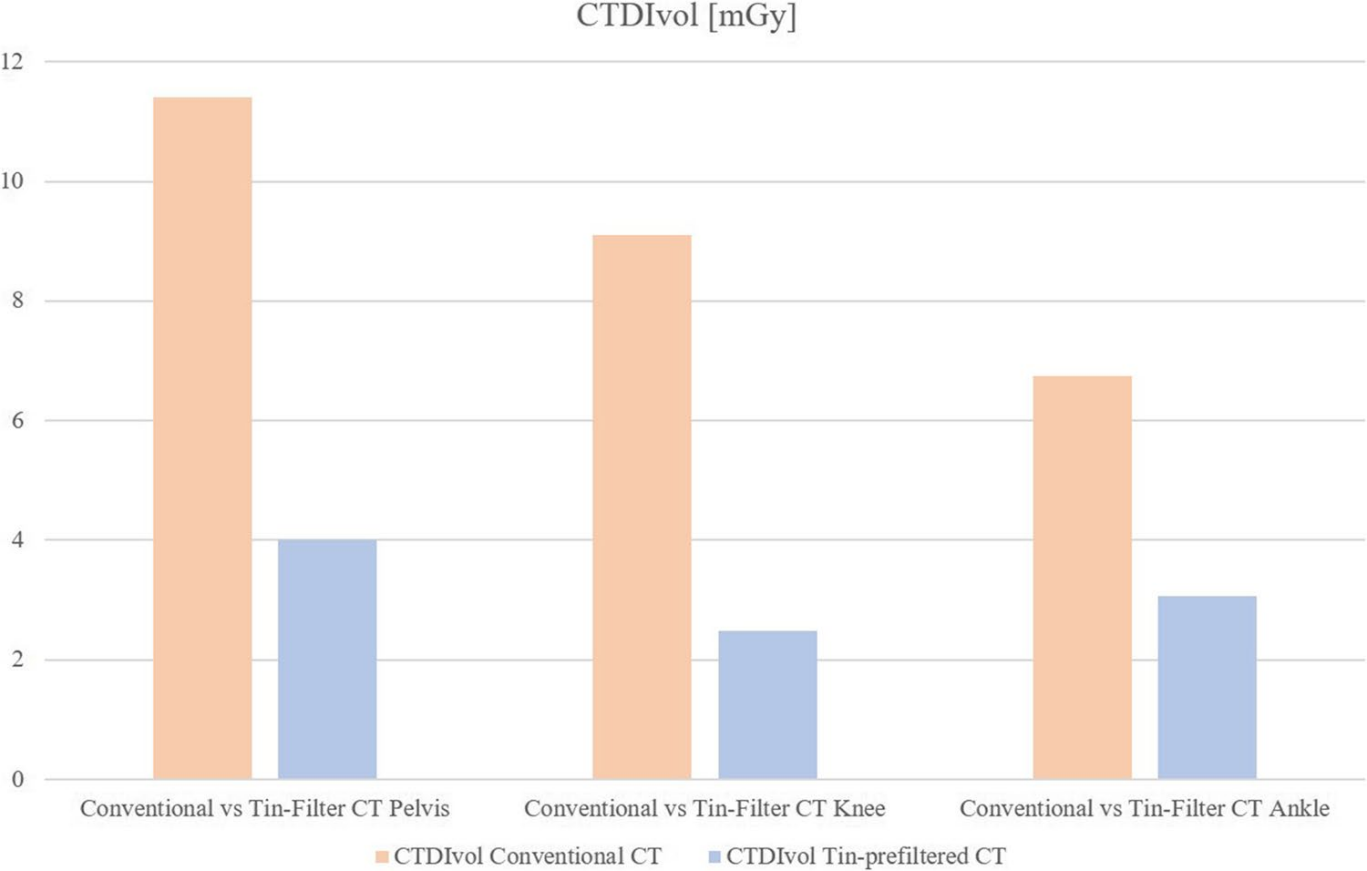
The median CTDI_vol_ of conventional (orange) and tin-prefiltered (blue) CT scans for the pelvis, knee, and ankle. Tin-prefiltered scans were conducted at a significantly lower CTDI_vol_, reducing the median by 65% in the pelvis, 73% in the knee, and 54% in the ankle

**Fig. 3:**
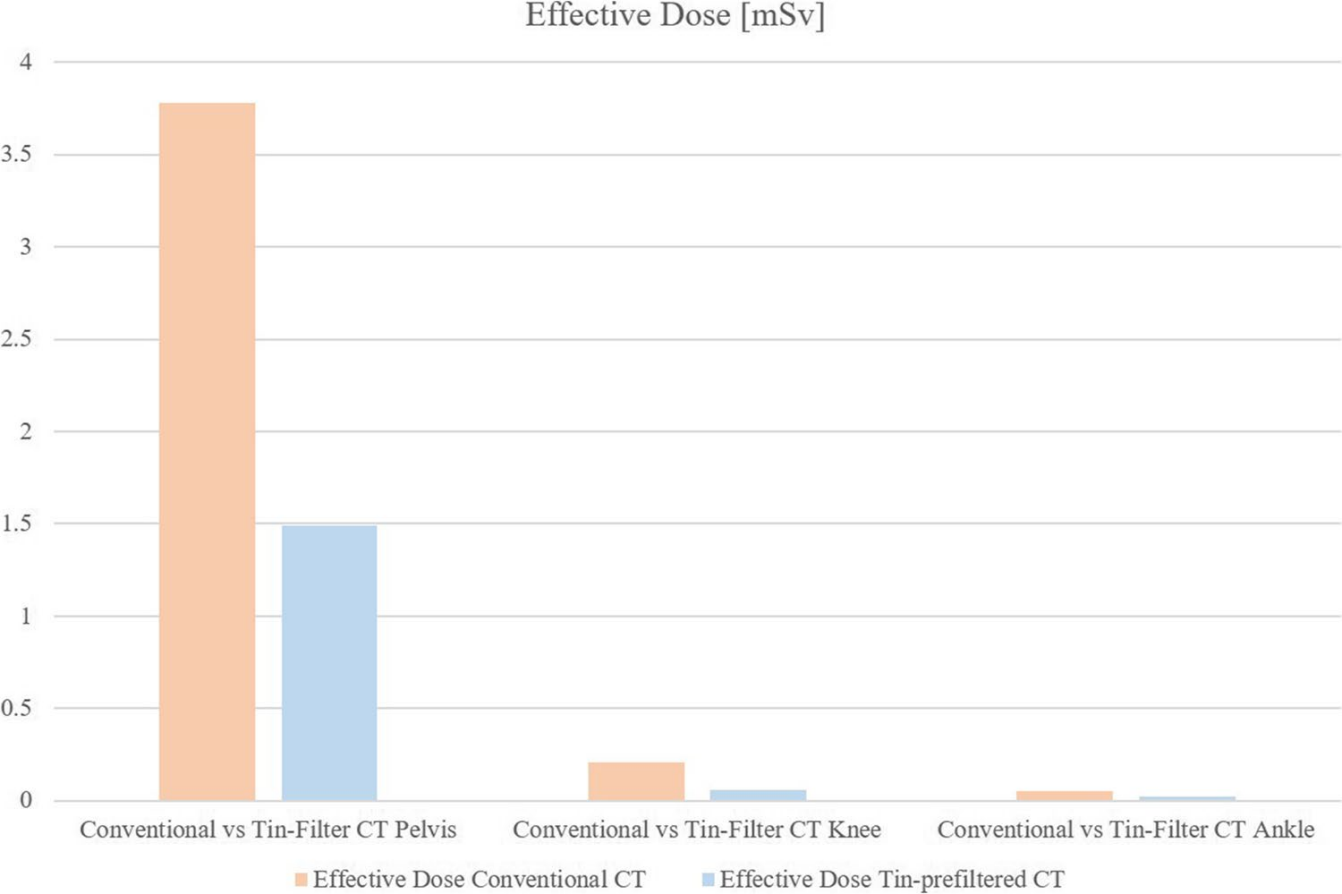
Calculated median effective dose of conventional (orange) and tin-prefiltered CT scans (blue). Tin-prefiltered CT scans resulted in significantly lower radiation doses, in specific, 61% in the pelvis, 71% in the knee, and 60% in the ankle, despite higher scan length in the pelvis (32%) and the knee (2%)

The CTDI_vol_, DLP, and effective dose were reduced significantly in all tin-prefiltered examinations compared to the conventional, non-tin-prefiltered examinations: Tin-prefiltered CT scans were conducted at a 65% lower median CTDI_vol_ in the pelvis (*p* < 0.001; reduction from 11.4 to 4 mGy), at 73% in the knee (*p* < 0.001; reduction from 9.1 to 2.5 mGy), and at 54% in the ankle (*p* < 0.001; reduction from 6.75 to 3.1 mGy). Despite the scan length being significantly higher in the tin-prefiltered scans of the pelvis and knee than in the conventional scans (both *p* < 0.001; pelvis 243.5 (226–261.2) mm vs. 322 (310–337) mm (+ 32%), knee 375 (375–376) mm vs. 381 (381–382) mm (+ 2%)), the DLP and effective dose were still significantly lower in the tin-prefiltered scans. The reduction of the median effective dose for the pelvis was 61% (*p* < 0.001); in absolute numbers from 3.78 to 1.49 mSv (− 2.29 mSv). For the knee scans, the reduction of the median effective dose was 71%, from 0.21 to 0.06 mSv (*p* < 0.001; − 0.15 mSv). No significant difference in scan length was seen in the ankle (*p* = 0.31), while the reduction in DLP and the effective dose was also significant for the ankle: median effective dose decreased by 60%, from 0.05 to 0.02 mSv (*p* < 0.001; − 0.03 mSv). All radiation dose parameters are reported in Table 3. The median CTDI_vol_ of the evaluated 50 conventional and 50 tin-prefiltered CT scans for the pelvis, the knee, and the ankle is displayed in Fig. 2; the median effective dose is shown in Fig. 3.

**Table 3:** CT radiation dose parameters

Pelvis	Conventional CT	Tin-prefiltered CT	*p*	Difference (%)
CTDI_vol_ (mGy)	11.4	4	< 0.001	65%
DLP (mGy·cm)	263.4 (244–282.4)	117.5 (112.4–123.5)	< 0.001	57%
Effective dose (mSv)	3.78 (3.65–3.89)	1.49 (1.47–1.50)	< 0.001	61%
Knee	Conventional CT	Tin-prefiltered CT	*p*	Difference (%)
CTDI_vol_ (mGy)	9.1	2.5 (2.4–2.6)	< 0.001	73%
DLP (mGy·cm)	330.4 (329.2–331.1)	89.5 (85.4–94)	< 0.001	73%
Effective dose (mSv)	0.21 (0.21–0.23)	0.06 (0.06–0.06)	< 0.001	71%
Ankle	Conventional CT	Tin-prefiltered CT	*p*	Difference (%)
CTDI_vol_ (mGy)	6.75 (6.6–6.9)	3.1 (3.0–3.1)	< 0.001	54%
DLP (mGy·cm)	115.4 (105–122.5)	50 (47.6–59.6)	< 0.001	57%
Effective dose (mSv)	0.05 (0.05–0.05)	0.02 (0.02–0.03)	< 0.001	60%

Values are reported as median, with the 25th percentile (Q1) and 75th percentile (Q3) in parentheses (Q1–Q3). The fourth column reports the percentage difference in the median of CTDIvol, DLP, and effective dose between tin-prefiltered CT and conventional CT

### Qualitative image analysis

**Fig. 4:**
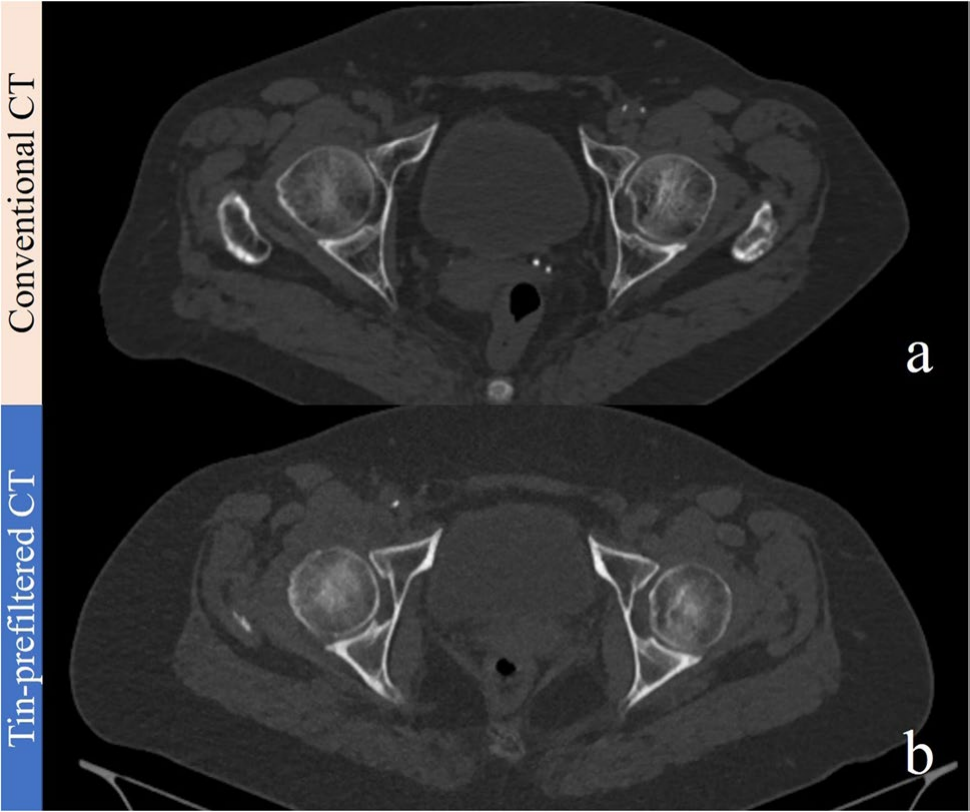
Conventional CT scan of the pelvis of a 71-year-old female (BMI 26.6 kg/m^2^) (**a**) compared to a tin-prefiltered CT scan of the pelvis of a 61-year-old female (BMI 26.6 kg/m.^2^) (**b**). CTDI_vol_ of the tin-prefiltered scan was 4.04 mGy compared to 11.3 mGy of the conventional CT scan. All images were reconstructed with the convolution kernel BR38, slice thickness of 1 mm, iterative reconstruction strength level 3, and displayed at a window/center level of 1500/450

**Fig. 5:**
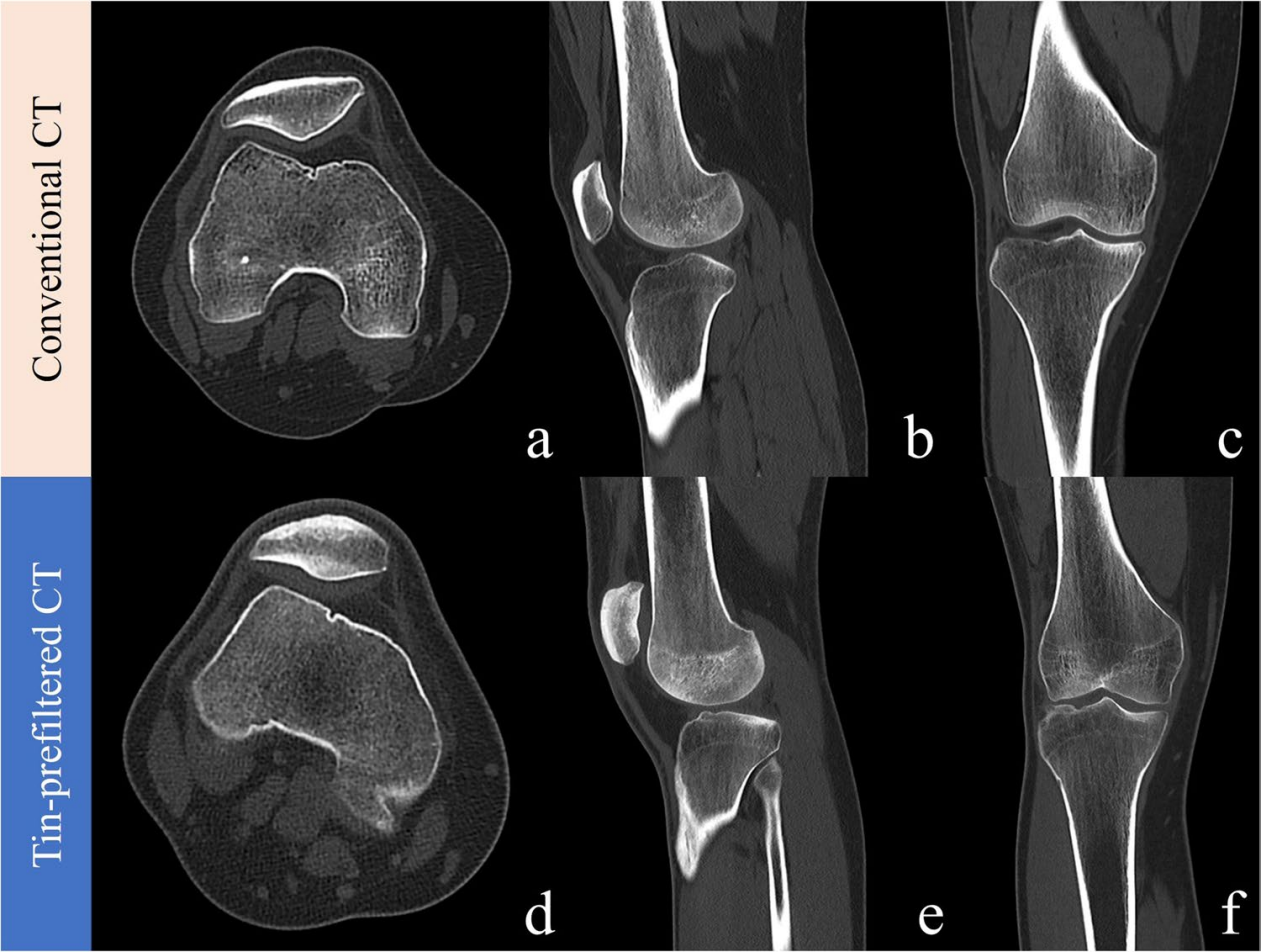
Conventional CT scan of the knee of a 26-year-old female (BMI 25.0 kg/m^2^) (**a, b, c**) compared to a tin-prefiltered CT scan of the knee of a 22-year-old man (BMI 24.9 kg/m^2^) (**d, e, f**). CTDI_vol_ of the tin-prefiltered scan was 2.59 mGy compared to 9.06 mGy of the conventional CT scan. All images were reconstructed with the convolution kernel BR59, iterative reconstruction strength level 3, and displayed at a window/center level of 1500/450. Axial images were reconstructed with a slice thickness of 1 mm, sagittal and coronal images at 2 mm slice thickness

**Fig. 6:**
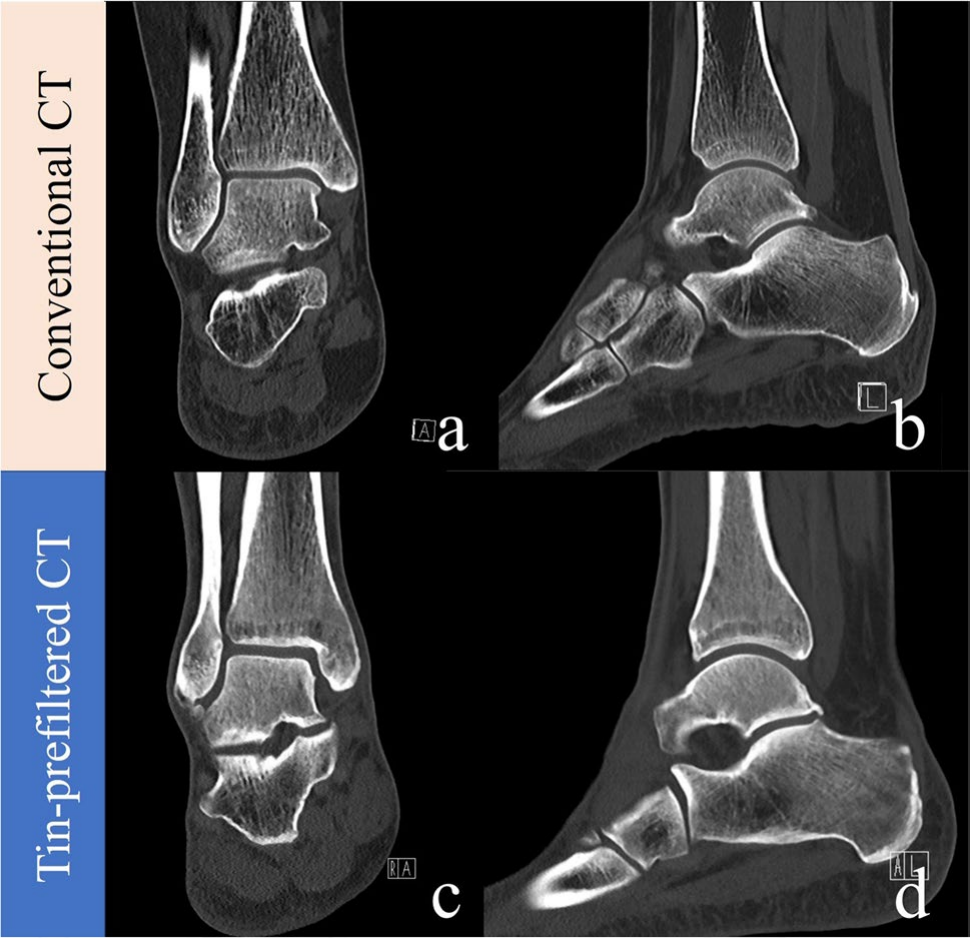
Conventional CT scan (**a, b**) of the ankle of a 38-year-old female (BMI 24.6 kg/m^2^) compared to a tin-prefiltered CT scan (**c, d**) of the ankle of a 39-year man (BMI 24.6 kg/m^2^). CTDI_vol_ of the tin-prefiltered scan was 3.07 mGy compared to 6.87 mGy in the conventional CT scan. All images were reconstructed with the convolution kernel BR62, iterative reconstruction strength level 3, and displayed at a window/center level of 1500/450. Both coronal images have a slice thickness of 1 mm, and both sagittal images have a slice thickness of 2 mm

Table 4 summarizes the results of the qualitative image analysis. Conventional and tin-prefiltered CT showed no significant difference in terms of any qualitative measure for any reader (all *p* > 0.05) in the knee and the ankle. The only significant difference in image quality was observed by both readers regarding image noise in the hip (both *p* < 0.001), with the median image noise being 4 (minimal) in conventional images and 3 (moderate) in tin-prefiltered examinations. The diagnostic image quality was 4 (good) for all tin-prefiltered and all conventional CT images for all of the three scan locations. No examination in either group received a score of 2 for any qualitative measure. Figures 4, 5, and 6 show representative images for BMI-matched patients in the two groups. Interreader agreement was substantial or almost perfect for all qualitative measures (κ = 0.77–1.00), as summarized in Table 5 (supplementary material).

**Table 4:** Qualitative image assessment

Feature	Reader 1			Reader 2		
	Conventional CT	Tin-prefiltered CT	*p*	Conventional CT	Tin-prefiltered CT	*p*
Pelvis						
Depiction of bone anatomy	4 (4–4)	4 (4–4)	1	4 (4–4)	4 (4–4)	1
Image noise	4 (4–4)	3 (3–3)	< 0.001	4 (4–4)	3 (3–3)	< 0.001
Image artifacts	4 (4–4)	4 (4–4)	0.31	4 (4–4)	4 (4–4)	0.24
Soft tissue	3 (3–3)	3 (3–3)	0.48	3 (3–3)	3 (3–3)	0.57
Diagnostic image quality	4 (4–4)	4 (4–4)	1	4 (4–4)	4 (4–4)	1
Knee						
Depiction of bone anatomy	4 (4–4)	4 (4–4)	1	4 (4–4)	4 (4–4)	1
Image noise	4 (4–4)	4 (4–4)	0.33	4 (4–4)	4 (4–4)	0.43
Image artifacts	4 (3–4)	4 (3–4)	0.09	4 (3–4)	4 (3–4)	0.24
Soft tissue	3 (3–3)	3 (3–3)	0.45	3 (3–3)	3 (3–3)	0.26
Diagnostic image quality	4 (4–4)	4 (4–4)	1	4 (4–4)	4 (4–4)	1
Ankle						
Depiction of bone anatomy	4 (4–4)	4 (4–4)	0.08	4 (4–4)	4 (4–4)	0.16
Image noise	4 (4–4)	4 (4–4)	0.32	4 (4–4)	4 (4–4)	0.32
Image artifacts	4 (4–4)	4 (4–4)	0.32	4 (4–4)	4 (4–4)	1
Soft tissue	3 (3–3)	3 (3–3)	0.43	3 (3–3)	3 (3–3)	0.35
Diagnostic image quality	4 (4–4)	4 (4–4)	0.56	4 (4–4)	4 (4–4)	0.56

Note—data are reported as median with the 25th percentile (Q1) and 75th percentile (Q3) in parentheses (Q1–Q3). The grading system used a 4-point Likert scale for the depiction of the bone anatomy, the soft tissue, and the diagnostic image quality (1 = poor, 2 = fair, 3 = moderate, 4 = good), the image noise (1 = very high, 2 = high, 3 = moderate, 4 = minimal), and for the image artifacts (1 = very strong, 2 = strong, 3 = weak, 4 = none)

### Quantitative image analysis

Image noise, defined as the standard deviation in the air, was significantly higher in the pelvis in tin-prefiltered images 12.7 (9.2–14.6) compared to conventional images 8.9 (7–10.8), *p* < 0.001. In the knee and ankle, image noise showed no significant difference in tin-prefiltered CT (knee 27.6 (25.7–32.3); ankle 24.3 (22.5–25.7)), compared to conventional CT (knee 28 (24.5–32.2); ankle 24.2 (22.5–25.7)), *p* = 0.68 and *p* = 0.46, respectively. SD in subcutaneous fat, often also used as a reference parameter for image noise, showed the same significant difference in the hip with higher image noise in tin-prefiltered examinations (*p* < 0.001), while no significant difference was observed in the knee and the ankle, *p* = 0.09 and *p* = 0.58, respectively. Conventional and tin-prefiltered CT values significantly differed for cortical bone, muscle, and subcutaneous fat (*p* < 0.05). The median CT value for cortical bone was significantly higher in conventional CT (pelvis 1455 HU, knee 1548 HU, and ankle 1797 HU) than in tin-prefiltered CT (pelvis 1099 HU, knee 1420 HU, and ankle 1568 HU). Also, CT values for muscle were significantly higher in conventional CT than in tin-prefiltered CT, while CT values of subcutaneous fat were significantly lower. Values are displayed in Table 6 (supplementary material). SNR for cortical bone, muscle, and subcutaneous fat was significantly higher in conventional examinations than in tin-prefiltered images in all three anatomical regions (all *p* < 0.05). The median SNR for cortical bone in conventional examinations in the pelvis was 163.8 (134.4–210.6 HU), for muscle 7.2 (5.2–8.4), and for subcutaneous fat 11.3 (8.9–14.6). In tin-prefiltered CT, the SNR in the pelvis was 88.9 (74.1–118.2) for cortical bone, 4.4 (3.9–6.3) for muscle, and 7.8 (6.5–9.2) for subcutaneous fat. CNR differed between tissues and anatomical regions and is displayed in Table 6 (supplementary table). In the pelvis, CNR was significantly higher in conventional CT (*p* < 0.05), CNRbone-muscle 47.7 (38.7–64.2), and CNRmuscle-fat 11.5 (9.9–14.7) than in tin-prefiltered examinations, 36 (29–45.9) and 7.8 (6.3–9.9), respectively.

## Discussion

The implementation of tin-prefiltration in clinical routine scan protocols significantly reduced the effective radiation dose for unenhanced CT scans of the pelvis (61% reduction), the knee (71% reduction), and the ankle (60% reduction).

While several studies have reported the potential of radiation dose reduction for anatomical structures with high intrinsic contrast, such as lung or bone, in a study setting [9, 11, 18–20], this is the first study reporting on the impact of tin-prefiltration on radiation dose for the musculoskeletal unenhanced CT protocols in clinical routine. When tin-prefiltration was implemented on our scanner to reduce radiation dose, the protocol optimization to maintain clinical image quality took several months and led to a specific adaptation of scan parameters for each scan protocol, particularly of kV and mAs, based on subjective image quality assessment. Three different joints of the lower extremities were included in this study to evaluate the effect of tin-prefiltration for different anatomic regions in a clinical setting. Scans with metal implants were excluded to avoid the influence of different compositions and geometries on radiation dose and image quality [21, 22].

The results showed a significant radiation dose reduction in clinical routine CT scanning of the pelvis, knee, and ankle since tin-prefiltration was implemented. While in absolute numbers the median dose and dose reduction in a single scan for the knee (0.06; − 0.15 mSv) and ankle (0.02; − 0.03 mSv) may be low, the median effective dose and dose reduction estimation are much higher in the pelvis (1.49 mSv; − 2.29 mSv). The main difference in the effective dose between the pelvis and the knee/ankle scans results from the different radiation sensitivity of the exposed tissues for the specific scan region. While in the pelvis radiation-sensitive organs like the bladder and gonads are exposed, in the knee and ankle, less radiation-sensitive tissues such as the skin, bone, and muscle, are exposed, which are less sensitive to the induction of stochastic effects [2]. To put these numbers in relation, it is helpful to look at the average annual background and medical radiation dose exposure per person per year. In the swiss population, the average annual radiation dose exposure is reported at 6 mSv, with medical imaging accounting for 1.49 mSv, and the annual background radiation being 4.51 mSv. In the USA, the annual radiation dose exposure is 6.3 mSv, with the medical radiation dose accounting for 3.0 mSv and the natural background radiation dose being 3.1 mSv [5, 7]. In relation to this, we achieved an absolute effective dose reduction of 2.29 mSv in a single pelvis scan. While the higher absolute dose reduction in the pelvis might send a clearer signal to implement tin-prefiltration in daily routine scans of the musculoskeletal system, also the lower absolute dose reduction in the knee and ankle scans should be considered, as international radiation protection authorities still rely on the assumptions of a linear no-threshold dose–response relationship for stochastic radiation dose effects. Although, the risk at low dose levels is classified as minimal at 0.1–1 mSv to negligible at < 0.1 mSv by the ICRP [23]. This is the first retrospective study reporting on the impact of tin-prefiltration on radiation dose reduction in musculoskeletal CT of the lower extremities in a clinical setting, but our results align well with the dose reduction of the tin filter reported in a research setting: Stern et al. evaluated unenhanced tin-prefiltered scans in the pelvis and demonstrated the feasibility of an ultra-low dose protocol with radiograph-equivalent radiation dose. With a 6.1-fold dose reduction compared to their standard clinical CT of the pelvis, the median effective dose was 0.38 mSv, a lower value than the median in our study with 1.49 mSv [10]. This suggests that a further dose reduction of our clinical scan protocol may be feasible for the depiction of bone anatomy and the detection of osseous pathologies, even though this may come with some decrease in image quality. Subjective image noise was rated lower in conventional CT scans than in tin-prefiltered scans in our study, which was also reported by Stern et al. when they compared the ultra-low dose tin-prefiltered scans to the higher dose clinical scans. As for diagnostic image quality, bone anatomy, image artifacts, and soft tissue, no difference was observed in our study between conventional CT scans and tin-prefiltered CT, and image quality was 4 (good) in all examinations. In accordance with subjective assessments, image noise was significantly higher in the quantitative parameters in the hip, while no significant difference in the knee and the ankle was observed. The quantitative assessment aligns with Stern et al., showing higher noise in tin-prefiltered low-dose images and higher SNR and CNR in conventional scans. Although these quantitative image parameters show significant differences, they did not influence diagnostic image quality, and significant differences in these objective parameters must be interpreted with caution. The results align also with the results obtained by another study by Stern et al. focused on tin-prefiltered CT imaging for lumbar spinal instrumentation. Tin-filtered CT saved 60% of radiation dose compared to standard CT with good image quality while performing superior regarding the width and attenuation of hypodense metal artifacts [24]. Suntharalingam et al. demonstrated in whole-body ultra-low dose CT with Sn 100 kV using spectral shaping in multiple myeloma sufficient image quality for depicting osteolytic lesions while reducing the radiation dose by approximately 74% [25].

In a phantom and retrospective clinical study setting, Schüle et al. demonstrated the potential of low-dose tin-prefiltered CT scans in follow-up pelvic imaging in patients with and without metal implants [8]. In the phantom, they showed that low-dose tin-prefiltered protocols (with a dose reduction of 67%) are equal to standard dose protocols for musculoskeletal imaging of the pelvis region. In the retrospective study with 45 enrolled patients with and without metal implants, only mild impairment in tin-prefiltered low-dose CT images with a 90% dose reduction to normal dose CT images could be observed due to metal artifacts, and the assessability of cancellous bone, but clinical questions could be answered without impairment. The low-dose tin-prefiltered pelvis protocol was conducted at a CTDI_vol_ from 1.3 to 2.6 mGy, with an effective dose of 0.5 to 1.1 mSv, being lower than in our study. As observed in our study for subjective image noise, the quantitative image noise in their study resulted in higher image noise compared with normal dose protocols.

Several limitations of our study have to be considered: effective dose, either calculated with conversion factors or advanced technologies, with Monte Carlo simulation and tested phantoms, or through VirtualDoseCT, are not exact values but remain an estimation, even though the impact on the study results are probably minimal. When the tin filter was installed on the scanner, the collimation was increased, which is known to increase dose efficiency. Dose efficiency was increased by an estimated 4%, i.e., from 92% in the conventional scans with a collimation of 32 × 0.6 mm to 96% in the tin-prefiltered scans with a collimation of 64 × 0.6 mm [26]. The scan length has to be considered for the comparison between conventional CT and tin-prefiltered CT, as it directly influences the DLP and the effective dose: the longer scan length in the pelvis and the knee in the tin-prefiltered scans of our study suggests an even higher dose reduction potential for tin-prefiltration if the scan length had been identical between conventional CT and tin-prefiltered CT. As automatic tube current modulation is a tool for reducing radiation dose, we preferred to compare examinations with the same modulation type, as in the pelvis (none) and the ankle (angular modulation). Comparing examinations of the same modulation type in the knee was not possible in our retrospective study setup. Excluding patients with orthopedic implants may have reduced the average patient age and the BMI, which may be associated with a more efficient x-ray utilization for CT-imaging, but outweighs limitations that would be introduced by implants of different orientation, geometry, and composition [21].

This study is the first to report the impact of the tin filter in clinical routine scans of musculoskeletal imaging of the lower extremities: The estimated dose saving was 61% in the pelvis, 71% in the knee, and 60% in the ankle. In accordance with the ALARA principle and no-threshold models for stochastic effects of ionizing radiation, the results support the use of tin-prefiltration in routine musculoskeletal CT for clinical examinations.

## Supplementary Information

Below is the link to the electronic supplementary material.

Supplementary file1 (DOCX 39 KB)

## Data Availability

Upon request, authors can send documentation or raw data in order to verify the validity of their results.

## Abbreviations

ALARA: As low as reasonably achievable
BMI: Body mass index
CNR: Contrast-to-noise ratio
CT: Computed tomography
CTDI_vol_: Computed tomography dose index volume
DLP: Dose length product
kV: Kilovolt
mAs: Milliampere-seconds
Q1: 25Th percentile
Q3: 75Th percentile
ROI: Region of interest
SD: Standard deviation
SNR: Signal-to-noise ratio
