# Supplementary material for: Implementing tin-prefiltration in routine clinical CT scans of the lower extremity: impact on radiation dose
Source: Skeletal Radiol. 2025 Feb 26;54(9):1915–25. doi: 10.1007/s00256-025-04897-3 (PMC12241252; doi:10.1007/s00256-025-04897-3)
Supplement: Supplementary file 1 — Supplementary file1 (DOCX 39 KB) [file 256_2025_4897_MOESM1_ESM.docx]

Supplementary material

|  |  |
| --- | --- |
| Feature | Agreement |
| Depiction of Bone Anatomy | 0.80 (0.41 - 1.00) |
| Image Noise | 0.84 (0.76 - 0.91) |
| Image Artifact | 0.77 (0.66 - 0.87) |
| Soft Tissue | 0.79 (0.69 – 0.87) |
| Diagnostic Image Quality | 1.00 (1.00 - 1.00) |

**Table 5** Interreader Agreement

Note - Data are expressed as weighted Kappa coefficient with 95% CI in parentheses. The agreement was categorized according to the system of Landis and Koch: less than 0.200, slight agreement; 0.200-0.399, fair; 0.400-0.599, moderate; 0.600-0.799, substantial; and 0.800 or greater, almost perfect.

|  |  | SD Air | HU Bone | HU Muscle | HU Fat | SD Bone | SD Muscle | SD Fat | SNR Bone | SNR Muscle | SNR Fat | CNR  Bone-Muscle | CNR  Muscle-Fat |
| --- | --- | --- | --- | --- | --- | --- | --- | --- | --- | --- | --- | --- | --- |
| *Pelvis* |  |  |  |  |  |  |  |  |  |  |  |  |  |
| Conventional CT | Median | 8.9 | 1455 | 59.9 | -100.9 | 28.9 | 13.7 | 10.2 | 163.8 | 7.2 | 11.3 | 47.7 | 11.5 |
|  | Q1 | 7 | 1389 | 55.8 | -96.1 | 21.6 | 10.8 | 8.6 | 134.4 | 5.2 | 8.9 | 38.7 | 9.9 |
|  | Q3 | 10.8 | 1506 | 65 | -109.5 | 35.9 | 16.8 | 14 | 210.6 | 8.4 | 14.6 | 64.2 | 14.7 |
| Tin-prefiltered CT | Median | 12.7 | 1099 | 55.7 | -95.2 | 29.4 | 19.3 | 17.1 | 88.9 | 4.4 | 7.8 | 36 | 7.8 |
|  | Q1 | 9.2 | 1064 | 51.8 | -89.6 | 23.1 | 15.8 | 13.5 | 74.1 | 3.9 | 6.5 | 29 | 6.3 |
|  | Q3 | 14.6 | 1143 | 62.9 | -102.4 | 36.3 | 24.4 | 22.6 | 118.2 | 6.3 | 9.2 | 45.9 | 9.9 |
|  | *p* | <.001 | <.001 | 0.021 | 0.002 | 0.986 | <.001 | <.001 | <.001 | <.001 | <.001 | <.001 | <.001 |
| *Knee* |  |  |  |  |  |  |  |  |  |  |  |  |  |
| Conventional CT | Median | 28 | 1548 | 66.1 | -117.5 | 136 | 53 | 51 | 56.8 | 2.4 | 4.2 | 10.7 | 3.4 |
|  | Q1 | 24.5 | 1500 | 62.3 | -111.9 | 117.5 | 46.6 | 42.5 | 46.6 | 1.9 | 3.9 | 9.7 | 3 |
|  | Q3 | 32.2 | 1598 | 71.2 | -122.5 | 153.7 | 60.5 | 59.5 | 64 | 2.9 | 4.7 | 12.5 | 3.9 |
| Tin-prefiltered CT | Median | 27.6 | 1420 | 63.7 | -104.9 | 100 | 47.2 | 45.9 | 51.5 | 2.2 | 3.7 | 13.2 | 3.5 |
|  | Q1 | 25.7 | 1386 | 56.5 | -101.1 | 88 | 42 | 40.8 | 43.6 | 1.9 | 3.4 | 11.5 | 3.1 |
|  | Q3 | 32.3 | 1467 | 66.3 | -109.2 | 118 | 56.1 | 54.5 | 55.7 | 2.4 | 4.1 | 14.9 | 3.8 |
|  | *p* | 0.464 | <.001 | 0.004 | <.001 | <.001 | 0.02 | 0.094 | 0.005 | 0.031 | <.001 | <.001 | 0.904 |
| *Ankle* |  |  |  |  |  |  |  |  |  |  |  |  |  |
| Conventional CT | Median | 24.2 | 1797 | 68.7 | -115.6 | 142.6 | 34.3 | 35.3 | 76.4 | 2.9 | 4.7 | 12.3 | 5.4 |
|  | Q1 | 21.9 | 1724 | 62.8 | -121.9 | 120.5 | 28.7 | 31.9 | 70.1 | 2.5 | 4.4 | 10.6 | 4.7 |
|  | Q3 | 25.9 | 1892 | 74.1 | -111.5 | 171.2 | 39.4 | 41.2 | 80.9 | 3.2 | 5.2 | 14 | 6.4 |
| Tin-prefiltered CT | Median | 24.3 | 1568 | 59 | -97.9 | 99.7 | 36.9 | 36.8 | 64.9 | 2.5 | 4 | 14.9 | 4.3 |
|  | Q1 | 22.5 | 1470 | 53.4 | -102.9 | 75.3 | 33.3 | 32.4 | 57.9 | 2.2 | 3.7 | 11.9 | 3.9 |
|  | Q3 | 25.7 | 1632 | 65.4 | -93.5 | 122.9 | 41.5 | 41 | 69 | 2.7 | 4.4 | 19.9 | 4.7 |
|  | *p* | 0.679 | <.001 | <.001 | <.001 | <.001 | 0.022 | 0.577 | <.001 | <.001 | <.001 | <.001 | <.001 |

**Table 6** Quantitative parameters

Note — Data are reported as median with the 25^th^ percentile (Q1) and 75^th^ percentile (Q3) in parentheses (Q1 – Q3). A *p*-value of < 0.05 was considered statistically significant between conventional and tin-prefiltered CT examinations.
